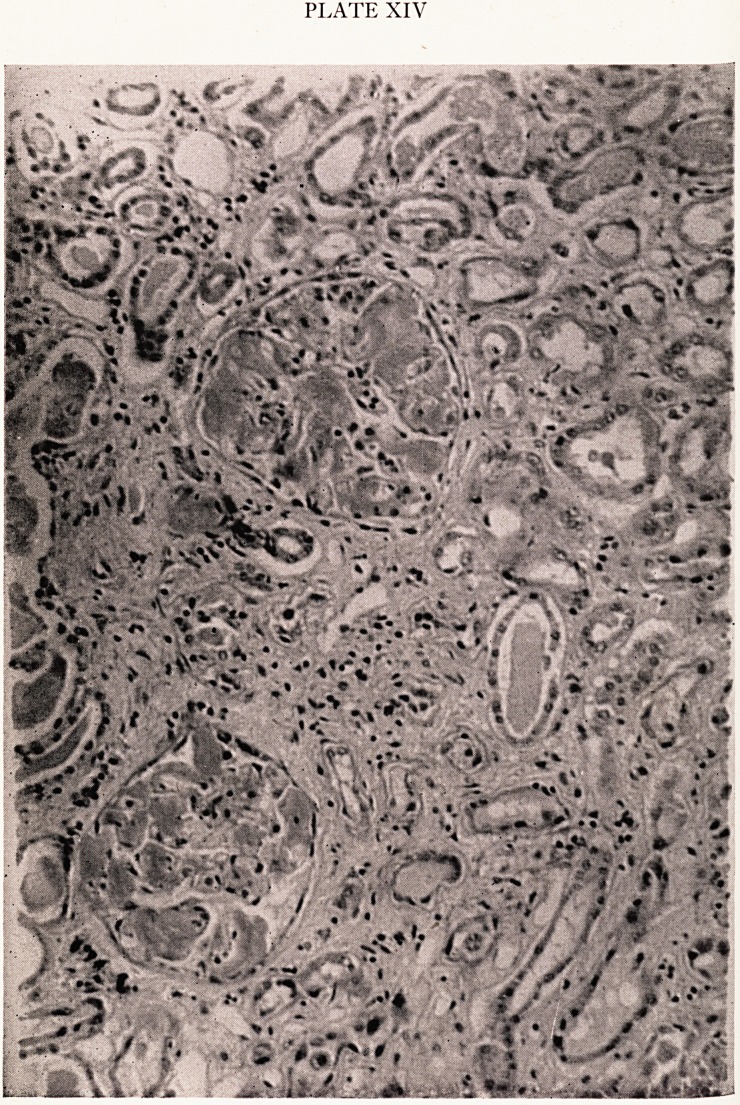# Nephrotic Syndrome with Steatorrhoea

**Published:** 1960-07

**Authors:** T. F. Hewer


### NEPHROTIC SYNDROME WITH STEATORRHOEA

*A Clinical Pathological Conference of the University of Bristol Medical School on 2nd February*, 1960

P.M.F. 273/59

CHAIRMAN: PROFESSOR T. F. HEWER

*Mr. K. D. J. Vowles:* This is the case of a 56-year-old unmarried Irishwoman, who was a cleaner and lived with her niece. She consulted her family doctor in August, 1959, because she had diarrhoea for two days, then two days of normal bowel habit, and then diarrhoea which was unremitting for five or six weeks. Her diarrhoea consisted of three or four loose, watery motions a day with an occasional drop of blood. Her family doctor sent her to Professor Milnes Walker's out-patients, here there was very little to be found on physical examination apart from a slightly distended and noisy abdomen. She was an extremely apprehensive woman, and the Professor felt that although the next step should be a sigmoidoscopy, this would have to be done in hospital under general anaesthesia. She was put on the waiting list for admission, and a barium enema was arranged meanwhile. This was reported as normal, though it did appear that the colon throughout its length had lost some of its normal distensibility.

She was admitted on 30th September, by which time she had had diarrhoea for six weeks and had lost 6 lb. in weight. She had no appetite and said that the had become increasingly nervy. On admission we saw a rather frightened, slightly wasted, pale woman. We were able to make out very little by ordinary physical examination: the abdomen was still slightly distended and rather noisy; rectal examination was negative. We didn't discover very much else except that she had a persistent tachycardia of 90 to 100 a minute, and during her ten days stay in this hospital she was midly febrile for most of the time. Two days after she came in I did a sigmoidoscopy under light general anaesthesia. I saw a normal anus, and a rectum which was rather pink and injected, and bled slightly when touched with the sigmoidoscope or a swab. There were no ulcers, there was no pus, and there appeared to be no mucus coming down from the bowel. I am afraid I have no very clear recollection of the character of the stool, if we saw any on that occasion; I certainly didn't make a note about it.

We felt that here was a woman in the older age group who was developing mild and unexplained ulcerative colitis, and that she should have medical treatment. Because we knew of Dr. Naish's special interest in the condition the Professor decided to consult him about her further care. At just about this time the house surgeon noticed that her blood pressure was persistently low, with a systolic pressure of 90 mm of mercury, and in view of this and of her tachycardia and continued diarrhoea we did her serum electrolytes and a blood count. Her haemoglobin was 71 per cent; her white count was 7,300, with a normal distribution of cells; her sedimentation rate was 80 mm in one hour and her serum electrolytes showed a normal sodium, a low potassium (2·65 mEq/Litre) and a low chloride (95 mEq/Litre); the serum protein also was low, the total being only 4·7 g per 100 mls. We felt that all these figures were adequately explained by her diarrhoea and by the condition we had seen on sigmoidoscopy. We started her on potassium supplements in the form of potassium citrate by mouth, and at that stage Dr. Naish saw her and took her to Frenchay Hospital for further investigation.

*Dr. Naish:* I was rather puzzled by this woman when I saw her at the Royal Infirmary. I didn't feel that she could be a straightforward case of ulcerative colitis for a number of reasons. Firstly, she had become ill so very quickly. Secondly, she had a very low serum potassium, which is unusual in a person who is only passing five or six motions a day, though extreme potassium loss may occur in people with much more severe diarrhoea. The third point was that she had some soft oedema of the ankles; I concluded that this oedema was due to protein loss because there was no evidence of heart failure, but I thought it unlikely that her diarrhoea was sufficient to cause hypoproteinaemia. The final point was that she had some finger clubbing, which I tend to associate with wasting disorders of small intestinal origin. Her abdomen was tumid, distended and gurgly on palpation. The whole clinical picutre looked to me more like a sprue syndrome than ulcerative colitis. On the other hand the occult blood test on the stools was positive, and Mr. Vowles had seen this rather red, granular mucosa in the rectum. I must confess that I was very puzzled as to the diagnosis: like many clinicians who can't make up their minds at once I decided to see more of the patient and think about her, and that is why she was transferred to Frenchay Hospital.

But it wasn't at all easy, even after further thought and investigation. We started by studying the protein metabolism, and we were rather staggered to find that her total serum proteins were down to 3·62 g per 100 ml and the albumin to the astonishingly low level of 0·146 g per 100 ml. I had noticed on the chart at the B.R.I. that albumin had been found in her urine on at least one occasion, and on checking this we found that she was excreting in her urine up to 10 g of protein a day. (Dr. Eastham will tell us something more of the detail of these biochemical investigations.) On sigmoidoscopy I noticed the same redness and granularity of the mucosa as was found by Mr. Vowles, but I was also able to see the stool coming down from above, and it was quite clearly fatty. One often finds a red, granular rectal mucosa in patients who are suffering from diarrhoea due to steatorrhoea; I presume that it is due to irritation of the mucosa by fatty acids. We found then that on a normal ward diet she was excreting an average of 9 g of fat a day, which is well above the normal figure. She had an anaemia, with a haemoglobin of 55 per cent and poor haemoglobinisation of the red cells. She was losing potassium in the urine as well as in the stools, and her serum potassium fell as soon as she was taken off her very high potassium diet. We reduced her potassium supplements for a short time, but only because Dr. McGowan, who was carrying out some special tests on her, asked us to do so.

So here was a lady who had become ill very rapidly, who had evidence of a malabsorption state and steatorrhoea, who was losing protein through her kidneys at quite a rapid rate, and whose serum albumin had fallen very low. This was a difficult thing to fit together, so we asked the radiologists if they could help us. We had in mind the possibility of a fistula between the small and large intestines, due to perforation of a diverticulum or perhaps through a small neoplasm between the two structures, since severe protein loss can occur in this condition. Dr. Gaskell accordingly made barium studies of the small intestine, about which he will tell you presently. The rest of her story was short. We tried to put protein into her intravenously as triple-strength plasma, but we found that the patient was losing protein from the bowel and kidney so fast that we just couldn't keep up with it; it was like trying to fill up a bath with a small cup when the plug is out. She had intravenous protein therapy for five days, but as we couldn't keep pace with the tremendous protein loss she weakened and finally died in a hypoproteinaemic state.

*Dr. Gaskell:* We have two lots of X-rays of this lady. The first examination was of the large intestine, carried out at the B.R.I. This did not show any special abnormality, but the radiologist conducting the examination noted that the calibre of the pelvic colon remaind rather narrow during the barium filling, which presumably signified an increase in tone. The films showed a normal colonic outline otherwise, and excluded the possibility of any fistulous connection with the small intestine. There was slight reflux through a rather incompetent ileo-caecal valve.

The second examination was conducted when the lady came to Frenchay and we were asked to do small intestine studies of her. Time did not allow us to start at the duodenum in this particular case, and we first saw her after she had been given half a cup of non-flocculable barium in the ward. Thereafter films at intervals showed rapid transit and dilution of the opaque material. Three hours after her meal there was segmentation of the barium, which had clumped into lumps, a feature indicating the presence of mucus. There was also a coarsening of the mucosal pattern, indicating swelling of the mucosa, which is generally due to oedema. The four hour film showed barium filling the large intestine round to the rectum; the transit was thus quite abnormally rapid.

What conclusions can we draw from this? Firstly, that there was a mucus-containing intestine showing abnormal structure and function, an appearance seen in steatorrhoea of the young or elderly and in tropical sprue, conditions described together for convenience under the heading of the malabsorption syndrome. Secondly, that there was no internal fistula to account for the diarrhoea.

Finally, a chest film was taken. From it you could see that the heart was displaced to the left, and a further density was visible through the heart shadow, which was reported as a collapsed left lower lobe. A second, more penetrating film did not define the area fully, but we assumed that the left lower lobe was indeed collapsed by some underlying disease of so far uncertain nature.

*Dr. Naish:* The lady had a loose cough which became worse during the fortnight or so she was at Frenchay, and we found a little dullness at the left base. I looked at these films and thought that possibly some of the opacity was due to fluid accumulating as part of her generalized anasarca.

*Dr. Eastham:* You have heard about the steatorrhoea. The upper limit of normal fat excretion in the stool, expressed as fatty acid, is 5 g per day, and she was excreting between 9 and 10 g per day, averaged over a period of three days.

She also had persistent proteinuria. Here (Fig. 1a) is the protein electrophoretic pattern of a normal serum. If you compare with this the pattern demonstrated in this patient's serum (Fig. 1c) you will observe the gross derangement, and in particular the complete absence of albumen. If now you compare the electrophoretic pattern of the patient's *urine* (Fig. 1b) with that of normal *serum* (Fig. 1a) a close resemblance is immediately obvious. This sample of urine was obtained after the patient had had a transfusion of double strength plasma. There was thus no doubt that we were dealing here with a nephrotic syndrome, whatever else might be there as well.

In an attempt to piece together the steatorrhoea and the nephrosis Dr. Naish suggested the possibility of lupus erythematosus or some other collagen disease. We examined the blood for LE cells without success, however.

There is not much more to say, except that over a five day period she lost about 10 g of protein each day in her urine. At the same time, over a ten-day period she received 22 bottles of plasma, equivalent to more than 500 g of protein. Since she remained grossly hypoproteinaemic despite this, with a serum protein level of about 3·6 g per 100 mls, it is evident that the kidneys were not the only route by which she was losing protein. It seems probable that the additional loss was via the alimentary tract.

It is perhaps worth mentioning that in addition to her total serum proteins of 3·6 g per 100 mls, the plasma contained 1·5 g of fibrinogen per 100 mls. Such high levels of fibrinogen occur in nephrosis.

*Dr. Naish:* A gram and a half is a fantastically high level.

*Dr. Eastham:* We noticed it at once because when the serum was sent for analysis it contained a large fibrin strand. Curiously enough the serum was not lactescent: in the nephrotic syndrome associated with glomerulonephritis the serum is usually milky, but this one was not.

*Dr. Naish:* Did you say anything about potassium loss?

| | | | | | |
|---|---|---|---|---|---|
| (*a*) | gamma | beta | alpha — 2 | alpha — 1 | albumin |
| (*b*) | gamma | beta | alpha — 2 | alpha — 1 | albumin |
| (*c*) | gamma | beta | alpha — 2 | Absent alpha — 1 and albumin | |

FIG. 1

(a) *Normal serum protein electropheresis pattern.*
(b) *Patient's urine protein electrophoresis pattern after plasma transfusion.* (*Note similarity to* 1 (a)).
(c) *Patient's serum protein electrophoresis pattern.* (*Note absence of alpha — 1 globulin and albumin*).

*Dr. Eastham:* On the few occasions when we estimated it we did not find much loss in the urine: the levels were of the order of 1 to 2 g per day, well within the normal range of up to 5 g per day. Since the serum potassium level tended to fall when she was taken off her high potassium diet one can only assume as you suggested, that it was being lost from the bowel.

*Professor Perry:* We were told originally that the albuminuria was inconstant. I was going to ask whether it became permanent, but it is obvious from what we have just heard that it did. With regard to the potassium loss, I believe that there is a syndrome in which such severe losses do occur from the intestine. Could Dr. Naish tell us whether the liver or spleen became enlarged?

*Dr. Naish:* The liver and spleen were never enlarged enough for us to feel them. Regarding the potassium loss from the intestine, this may of course be very great if there is diarrhoea originating high up in the intestine, as for instance in chronic pancreatitis or with a fistula between the large and small intestines. Even in ordinary adult coeliac disease you get quite a tendency to potassium depletion. It is quite uncommon, I think, in chronic ulcerative colitis unless there is terrific diarrhoea. There is of course a special syndrome of potassium loss associated with a papilliferous tumour of the colon.

*Professor Perry:* One other thing: is it really fair to assume that there must be some other protein loss, other than in the urine, if despite fairly massive plasma transfusion you can't raise the plasma proteins? My experience in cases of what I regard as perfectly simple, honest-to-goodness nephrosis is that this is just what happens. I have tried with three or four times concentrated plasma to raise their plasma proteins, but with little success, and I haven't been able to account for all of it in the urine. I have a feeling that there is somewhere in the body where you store protein, and that when that store is depleted you make it up first; but I'm afraid that it is just an idea. I wondered whether it was fair to say that because her blood protein failed to rise despite these plasma transfusions the protein given to her must have been lost.

*Dr. Naish:* It wasn't only for that reason. It was rather because the proteins had fallen so fantastically low in such a short time, and because we knew that she had a malabsorption state, that we thought it most likely that she was losing protein via the intestine as well.

*Professor Perry:* She was not absorbing her protein either, was she?

*Dr. Naish:* No, that's quite true. But attention has been focussed in recent years on the possibility that you can lose vast quantities of protein from the gastro-intestinal tract. It can happen with hypertrophic gastritis and in certain of the sprue syndromes.

Might I try to answer the question you asked earlier about the intermittency of the protein loss in the urine? I can't say anything about her state when she was in the B.R.I. because I have no charts. For the first two days after she came over to Frenchay the position is uncertain, but after we started looking for albuminuria regularly it was there all the time.

*Professor Perry:* I am really wondering whether something developed on top of her malabsorption syndrome which might have given rise to the massive albuminuria before she died.

*Dr. Naish:* It certainly looked like that.

*Professor Perry:* That was why I asked you about the liver and spleen.

*Dr. Eastham:* This table (Fig. 2) gives you briefly the percentages of albumin in the urine about the time when the intensive plasma transfusions were started. Before the transfusions no albumin could be detected in the urine, but after the transfusions

FIG. 2

| DATE | URINE | |
|---|---|---|
| | *Total protein* | *Albumin* |
| 30–31/10/59 | 7·22 g | nil |
| 31/10–1/11/59 | 6·28 g | nil |
| | Plasma transfusion started (4 bottles per day). | |
| 1–2/11/59 | 8·59 g | 19·3% |
| 2–3/11/59 | 14·5 g | 41·9% |
| 3–4/11/59 | 11·5 g | 48·5% |
| 5–6/11/59 | aliquot only received | 60·0% |

began the percentage of albumin in the urine rose steadily. I quite agree that we have not explained where all the protein was going. In the nephrotic syndrome the oedema fluid may increase the potential fluid volume into which the protein can pass from, shall we say, 3–5 litres to about 11 litres. Even so, after 22 bottles of plasma there are 500 g of protein to be explained away, and we have only accounted for about 40 g.

*Professor Hewer:* Could Dr. Naish tell us something about clubbing of the fingers in association with lesions of the small intestine?

*Dr. Naish:* People with adult coeliac disease, or non-tropical sprue, often have finger-clubbing, and they are a bit pigmented. It is quite a characteristic appearance, though not all such patients have it.

*Dr. Wright:* If I may add one small piece of information, I measured her total body potassium on three occasions. In spite of the fact that she had massive doses of potassium all the time she was in the B.R.I. and in Frenchay the total potassium fell throughout, and was in fact lower just before her death than when she was first seen.

*Dr. Naish:* The diseases which we considered as possibilities here, since they can cause both the disorder of the small intestine and the derangement of renal function, were disseminated lupus erythematosus (for which we could find no evidence at all) and amyloidosis. I should perhaps explain that this is one of the reasons why this desperately ill patient got away without any treatment with corticosteroids: they are said to make things worse in amyloidosis. It was really difficult to stand by and watch this lady just wastlng away, and to be able to do nothing for her except pour plasma in, and even this was eventually abandoned when it became clear that we could not help her.

*Professor Hewer:* Was there anything abnormal in the cellular content of the stools?

*Dr. Naish:* We usually ask for a wet film of the faeces to be examined to determine whether there is any excess of fatty acid crystals (typical of amlabsorption syndrome) or oil globules and meat fibres as in chronic pancreatitis. I can't find any record of such an examination in this case, I'm afraid.

*Dr. Sandry:* I had some warning of the complexities of this case before I was actually called into consultation, so to speak; the patient was a major topic of conversation in the laboratory for about a fortnight before she died. When I did eventually see her I found her to be a wasted woman, with slight oedema of the sacral tissues but none elsewhere. The serous cavities were moist, but not excessively so. The heart was small, weighing only 180 grammes: it showed some dilatation of the right and left ventricles but no other abnormality. The blood vessels were healthy, with one exception which I will mention presently. The kidneys were swollen, the left weighing 180 grammes and the right 185 grammes. Externally they appeared smooth and pale, the capsule stripping quite easily from each. The cut surfaces were pale, the pallor being evident principally in the cortex, which bulged somewhat. Having hear rumours of amyloidosis as well as simple honest-to-goodness nephrosis (a condition mentioned by an earlier speaker, which I do not personally recognize) I looked particularly carefully at the kidneys: it seemed to me that they did not present the picture of amyloidosis at all, the soft, bulging cut surface being quite unlike the waxy pallor of amyloidosis. The renal pelves, ureters and bladder were quite healthy. One tributary of the right renal vein in the upper pole of the kidney was partly occluded by mural thrombus; that part of the kidney did not appear any different from the rest, and I could not believe that the thrombus had really had any great effect on the circulation through it. The adrenals were slightly nodular and a little swollen, and showed some loss of cortical lipoid. The spleen also was rather swollen, weighing 195 grammes; it looked very engorged, with a rather softened pulp and possibly a little hyperplasia of the Malpighian bodies. The soft cut surface bulged slightly, and again did not present the appearance of amyloidosis at all. The liver was enlarged, weighing 2,050 grammes. To the naked eye it showed acute venous congestion with probably a good deal of fatty change; it had a rather greasy cut surface, but again not the waxy pallor of amyloid infiltration. The gall bladder and bile ducts were healthy and the pancreas appeared to the naked eye quite normal.

Turning now to the alimentary tract, the oesphagus and stomach appeared quite normal. The duodenum showed a little patchy mucosal congestion but nothing more. The small intestine also showed a little mucosal congestion, but in the autopsy room the mucosal pattern appeared quite normal; the contents were dark, greyish-brown and fluid. The caecum and appendix looked healthy. The colon appeared slightly thickened, its wall a little oedematous and its mucosal surface rather granular in appearance, but I could see no ulceration. The rectum was healthy. A few calcified mesenteric lymph nodes represented a healed abdominal primary tuberculous complex.

The right lung was congested and oedematous. In the lower lobe of the left lung (which had been the subject of a certain amount of speculation clinically) there was a large growth, 9·5 cm in diameter (Plate XIII). It occupied the basal segments of the lower lobe, and the main lower lobe bronchus ran into its centre. I found no metastases anywhere in the body. This tumour must I suppose have been hiding behind the (very small) heart. I found no change in the central nervous system.

I emerged from the autopsy room having decided that this was not a case of amyloidosis, but supposing that it must be nephrosis, though whether it was simple honest-to-goodness nephrosis I could not say. The first histological section, available a few days later, gave me no surprise: it showed the pulmonary tumour to be a rather poorly differentiated squamous carcinoma, extensively necrotic but without much in the way of inflammatory changes superimposed upon the necrosis. When I examined the first sections of the kidneys, however, I was mortified to discover that this was in fact a typical and severe example of amyloidosis. Here (Plate XIV) are two glomeruli showing amyloid deposition in the capillary tufts. Amyloid was also present beneath the basement membranes of many of the tubules, in the interstitial tissue throughout the cortex, and in the medulla as well. There was a little material giving the staining reactions of amyloid actually in some of the tubular epithelial cells, which throughout the whole kidney appeared rather flattened and degenerate. There was even similar material in the lumina of some of the tubules. The thrombus in the tributary of the right renal vein showed some organization and had evidently been present for some little time. The spleen showed amyloidosis of the nodular type; of course, on looking back at the fixed specimen I was able clearly to see the Malpighian bodies with their amyloid, greyish in colour, sago grains if you like that sort of thing. The liver contained a lot of fat and a very little amyloid, and showed considerable acute venous congestion; its enlargement appears to have been due principally to fatty change and acute congestion, and not to amyloidosis. As regards the other organs, the adrenals showed considerable though patchy amyloid deposition, chiefly between the cords of cortical cells in the zone fasciculata. The pancreas contained no obvious amyloid and showed no evidence of pancreatitis. The thyroid showed quite considerable amyloid deposition, a good deal of colloid storage and some scarring. The single parathyroid I examined contained a trace of amyloid only, not enough to alter its structure. The pituitary contained no amyloid, and I could not demonstrate amyloid in the myocardium.

I had hoped that study of the alimentary tract might be quite informative as the autopsy was only eight hours after death, but unfortunately there was considerable autolysis throughout the intestine. However, although the amyloid change is not striking, on close study one can see that there is in fact widespread deposition beneath the endothelium of the small blood vessels. I have examined sections from about a dozen different levels in the alimentary tract; there is some variation in amount at different levels, but there is obviously a great deal of amyloid present. There is a little lymphocytic and plasma cell infiltration in the submucosa and lamina propria mucosae of the small and large intestines, but I cannot see any actual ulceration. Accurate assessment of the degree of mucosal damage is impossible because of the severity of the autolysis: the clinical symptoms of persistent diarrhoea and the derangement of fat absorption suggest that there was a considerable functional disorder.

In summary, therefore, this turned out to be a case of severe amyloidosis, affecting principally the kidneys, spleen and alimentary tract, and associated with a bronchial carcinoma (you could perhaps call it an occult bronchial carcinoma), certainly necrotic but not apparently severely infected. The clinical presentation of the case seems to me to have been remarkable for several reasons, outstanding among which I find the acuteness of the onset of the initial symptom of diarrhoea, the steatorrhoea and the extraordinarily gross protein loss, particularly through the kidneys. This is not the time for a lecture on amyloidosis, so I will leave the matter there for discussion.

*Professor Hewer:* I don't suppose you examined the tongue for amyloid, did you?

*Dr. Sandry:* I don't know, sir, why you should say that you don't suppose I examined the tongue! You are quite right, of course, I didn't. It is one of the major deficiencies of my examination. I did examine the heart muscle, but I did not examine the tongue or the subcutaneous tissue. I would like to have examined the bone marrow: in fact, I shall do so, as I have recently found a fragment among the specimens, but I don't really think there is any possibility of myelomatosis in this case.

*Professor Hewer:* I expressed my question like that because I thought that as amyloidosis was only divulged to you later, it would have been very surprising if you had taken a section at the time.

*Professor Perry:* I am not clear whether this is put forward as a case of generalized amyloidosis secondary to a carcinoma of the bronchus, or as a case of primary amyloidosis with a coincidental carcinoma of the bronchus, or as a case of malabsorption syndrome and secondary amyloidosis with an incidental carcinoma of the bronchus.

*Dr. Sandry:* Of course I knew that somebody would ask this question. When I saw Professor Perry come into the room just now I had a shrewd idea who it would be. I personally would say that this should be regarded as a case of secondary amyloidosis in association with a bronchial carcinoma. I do not know how the carcinoma acted as an aetiological agent, but I do not think that anyone knows just how any of the recognized aetiological agents acts in producing this disorder. I would say that the malabsorption syndrome and the diarrhoea were secondary phenomena, essentially due to amyloid deposition in the bowel. Diarrhoea is a recognized, though not I think a very common symptom of amyloidosis. In regard to the steatorrhoea, I wonder in how many cases of diarrhoea due to amyloidosis the fat content of the stools has been estimated. I don't know whether steatorrhoea would be found commonly if this were done; it has been recorded, I think: in fact I'm sure it has. With regard to the distinction between primary and secondary amyloidosis, my impression, from an admittedly rather sketchy reading of the literature, is that there is a growing tendency to insist less upon the distinction between the two conditions nowadays. As Professor Symmers says in a recent article, although in amyloidosis without any obvious cause (commonly called primary) the substance tends to be distributed chiefly in the connective tissues rather than in the parenchymatous organs, and the reverse is true of amyloidosis in which an acceptable cause can be found (commonly called secondary), nevertheless one may find a typical "primary" distribution in a case with an obvious predisposing factor, and vice versa. It is in fact quite common to find severe involvement of the kidneys, spleen and liver in the so-called primary type of amyloidosis.

*Professor Hewer:* Yes, that is exactly what Symmers said. On the whole one expects to see the tongue and heart involved in the primary type, but it is not necessarily so. The albuminuria, of course, is not surprising in severe amyloid kidney; this was quite a severe grade of amyloid kidney, wasn't it?

*Dr. Sandry:* Yes.

*Professor Hewer:* All the glomeruli were very much involved. The nephrotic syndrome is a not infrequent result of amyloid kidney, and she was obviously losing a tremendous amount of albumin at the time, partly because of the amyloid kidneys. Having achieved a very low plasma albumin one would have expected her to develop a very high level of lipid in the blood.

*Dr. Sandry:* Yes, but in fact this did not appear to be so.

*Professor Hewer:* She was losing a great deal of albumin by some route which was not determined, more than could be accounted for by the renal leakage. It must undoubtedly have been escaping from the gut, in which there was severe amyloidosis. I wonder whether lipid could have been escaping as quickly as albumin was, from the gut.

*Dr. Eastham:* I don't know any actual work on faeces which has shown the pattern of the protein loss.

*Dr. Naish:* With regard to the frequency of steatorrhoea in amyloidosis, the association is well recognized, and one does occasionally come across a patient with a steatorrhoea syndrome who has amyloidosis of the small intestine and nowhere else. I remember an old chap at Snowdon Road who had steatorrhoea of uncertain origin. He was passing "aluminium-paint" stools. For a very long time we couldn't explain this state, but when he died the post-mortem examination revealed amyloidosis confined entirely to the small intestine. Another case I can call to mind was a patient who had tuberculosis and developed Addison's disease. We treated her with small doses of cortisone for two or three years, but finally she fell ill with diarrhoea and wasting and eventually died. The autopsy revealed amyloidosis not only of the intestine but also of the adrenals, and this was presumably the cause of her Addison's disease.

*Professor Hewer:* I probably missed this point in the biochemical story, but was her dietary fat intake measured, and if so was there any relation between this and the fat output in the stools?

*Dr. Naish:* No, this was not done. I should say that the lady was taking well under the average ward diet, which contains about 70 g of fat a day, for she had a very poor appetite and was very ill. She was losing about 9 g a day, which is not excessive: I have known a patient with acute pancreatitis who was losing up to 70 or 80 g of fat a day, strangely enough on a dietary intake which was less than that. It has been shown that there are fat-producing organisms in the bowel, so that measurement of the dietary fat intake is not very helpful.

*Professor Neale:* Actually I suppose that the steatorrhoea here, being mild, could have been due entirely to intestinal acceleration. I don't know if you said what the speed of transit through the gut was?

*Dr. Gaskell:* It was very erratic.

*Professor Neale:* Was the time factor actually worked out?

*Dr. Gaskell:* Yes, it was about three hours.

*Professor Neale:* Surely such a rapid transit fully explains the steatorrhoea, there being no time for absorption.

*Professor Perry:* The absence of lipoid in the serum is interesting. Five years ago some people were saying that the best test for steatorrhoea was to give a patient some fat and have a look at his plasma an hour to an hour and a half afterwards. I believe that this has not proved as satisfactory a test as had been hoped.

*Dr. Naish:* I know it is rather speculative at this distance, but I would not agree with Professor Neale that the malabsorption here was due purely to hurry through the intestine; I think it was much too severe. I have seen people who run barium through to the colon in about a couple of hours, very nervous characters with gurgly tummies, and they have no malabsorption. I was convinced, personally, that the loss of protein was taking place largely through the bowel, though it was occurring through the kidney as well. She had gone downhill so quickly.

*Dr. Gaskell:* No questions have been asked so far about the place of sepsis in this

PLATE XIII

case. We have not heard much about the blood picture, and Dr. Sandry has not told us how much sepsis there was distal to the growth in the bronchus.

*Dr. Sandry:* The growth showed a good deal of necrosis, but little evidence of inflammation. It extended to the pleural surface, so that there was little or no lung tissue beyond it in which sepsis due to bronchial obstruction might have occurred. In fact I was not able to demonstrate any severe sepsis anywhere.

*Professor Hewer:* Of course these squamous cell carcinomas can be very slowly growing. I wonder whether Dr. Gaskell thinks that this one could have been hiding behind the heart at the time of the chest X-ray, or whether it must have increased in size since then.

*Dr. Gaskell:* I feel certain that the tumour is in fact visible in the X-ray; there is a double density within half a centimeter of the cardiac border, which may be the actual edge of the tumour or the margin of the collapsed lower lobe. Such an abnormal finding is of course ample justification for further investigation.

*Professor Neale:* Could I ask one simple point? Do the findings in this case throw any light at all on the mechanism by which protein escapes into the urine in nephorsis? Here we have a demonstrable lesion, whereas I believe that in ordinary nephrosis there is very little that can be demonstrated histologically.

*Professor Hewer:* There are changes in the basement membrane of the glomeruli in such cases.

*Professor Neale:* Are there any comparable changes in this particular case? The end result is similar, loss of albumin in the urine. Is any light thrown upon the nephrotic albuminuria by the microanatomy (micropathology, if you like)?

*Dr. Sandry:* In this case, sir, the *ultra*-microanatomy was not studied: I have no access to an electron microscope.

*Professor Hewer:* I think we shall have to assume that the amyloid is responsible for most of these changes, though we do not really know why the amyloid is there. It is most unusual to find amyloidosis as a complication of a solitary pulmonary neoplasm of this kind.

*Professor Neale:* One final question. Is there only one type of amyloid material, or are there several different kinds?

*Professor Hewer:* There are several chemically distinct types.

*Professor Neale:* Can you establish that by differential staining?

*Professor Hewer:* The staining reactions show great variability from case to case.

### REFERENCE

Symmers, W. St. C. (1956). "Primary amyloidosis: A Review". *J. Clin. Path.*, **9**, 187.

## Figures and Tables

**Fig. 1 f1:**
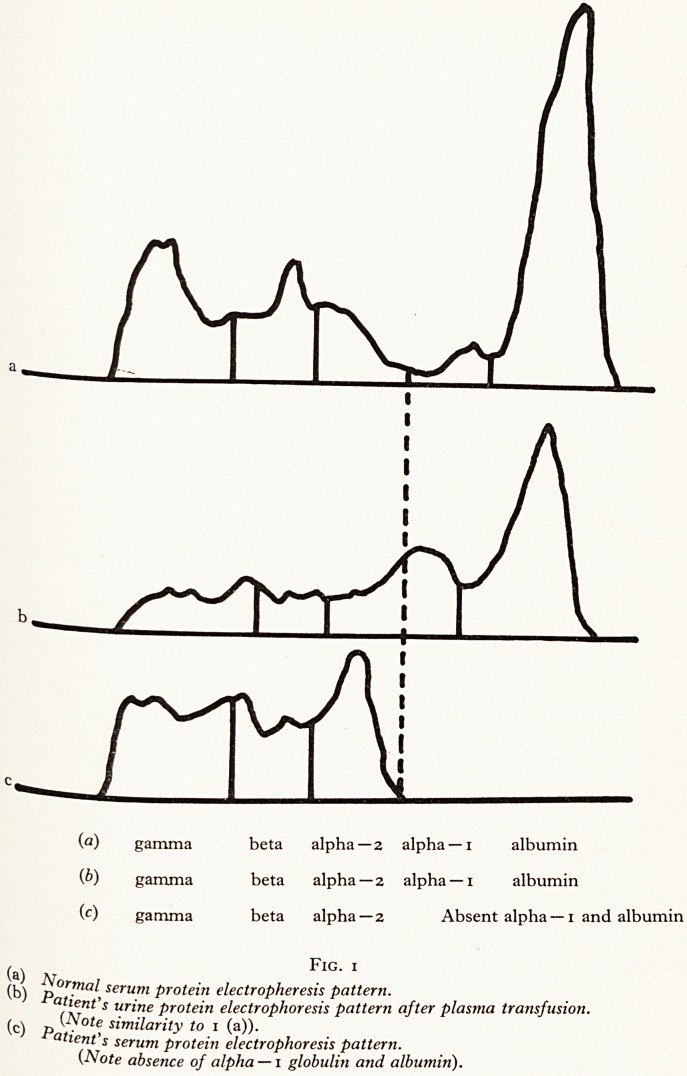


**Fig. 2 f2:**
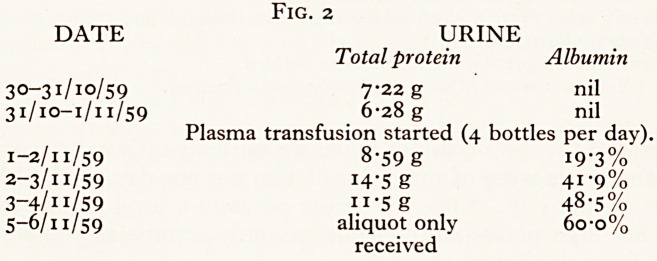


**PLATE XIII f3:**
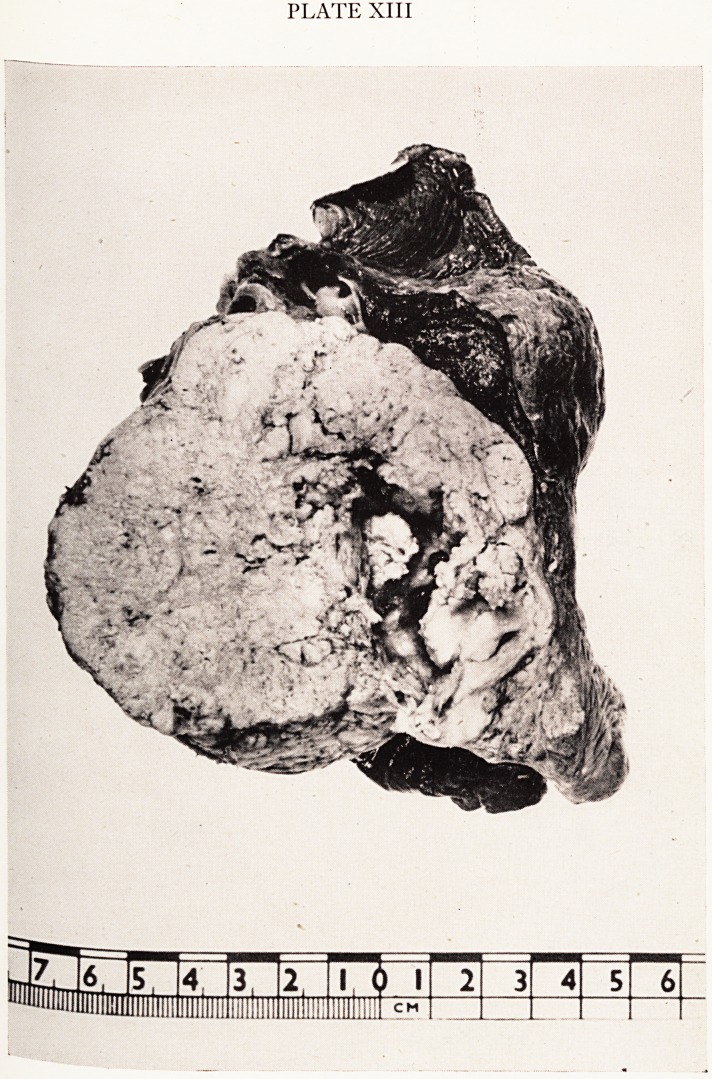


**PLATE XIV f4:**